# Immunohistochemical expression of P53 protein in nephroblastoma: a predictor of unfavorable prognosis

**DOI:** 10.1186/s43046-023-00183-2

**Published:** 2023-07-31

**Authors:** Emmanuel D. Morgan, James J. Yahaya, Advera I. Ngaiza, Emmanuel Othieno, Okwi A. Livex

**Affiliations:** 1 Department of Pathology, School of Health Sciences, Soroti University, Soroti, Uganda; 2 Department of Pathology, Muhimbili National Hospital, Dar-Es-Salaam, Tanzania; 3 Deparment of Pathology, Muhimbili University of Health and Allied Sciences, Dar-Es-Salaam, Tanzania; 4 Department of Pathology, Makerere University College of Health Sciences, Kampala, Uganda

**Keywords:** Nephroblastoma, P53, Immunohistochemical expression, Prognosis

## Abstract

**Objective:**

Immunohistochemical expression of P53 protein is so closely related to status of mutation of P53 gene which is tightly linked with pathogenesis of nephroblastoma or Wilms tumor. This study aims to determine the immunohistochemical expression of P53 protein and its predictors in formalin-fixed paraffin-embedded tissue blocks of patients with nephroblastoma.

**Materials and methods:**

A series of 83 histologically diagnosed cases of nephroblastoma from formalin-fixed paraffin-embedded tissue blocks archived at the Department of Pathology, Makerere University, in Kampala, Uganda, were analyzed. Monoclonal anti-p53 antibody (DO-7, DAKO) was used to assess the expression of P53 protein expression. Multivariable logistic regression analysis was performed to determine the predictors of P53 protein immunohistochemical expression, and statistical significance was considered when *p*-value was less than 0.05.

**Results:**

Most (42.2%, *n* = 35) of the cases were in advanced tumor stages (III–V), and almost one-quarter (21.7%, *n* = 18) of the cases were in high-risk group. The immunohistochemical expression of P53 protein was (8.4%, *n* = 7), and there were more (83.3%, *n* = 5) positive anaplastic cases for P53 protein compared with (2.6%, *n* = 2) of P53 expression for non-anaplastic cases. High risk (*AOR* = 3.42, 95% *CI* = 7.91–12.55, *p* = 0.037) and anaplasia (*AOR* = 1.41, 95% *CI* = 13.85–4.46, *p* = 0.001) were potential predictors of immunohistochemical expression of P53 protein.

**Conclusion:**

Most of patients with nephroblastoma in resources-limited settings are diagnosed with advanced clinical stages. Association of P53 protein with anaplasia found in this study indicates the possibility of having novel target therapy for treatment of patients with anaplastic form of nephroblastoma with a focus of identifying molecules that lead to its suppression in such subpopulations of patients with nephroblastoma.

## Introduction

Nephroblastoma or Wilms tumor (WT) is an embryonal type of renal cancer which histologically mimics renal embryogenesis [1]. Nephroblastoma consists of three components including blastemal (undifferentiated cells), epithelial (abortive tubules), and stromal (mesenchymal) component [2]. Nephroblastoma is the most common solid renal malignant tumor in children [3], and it comprises approximately 8% of all childhood cancers and 90% of pediatrics renal tumors [4]. Globally, the incidence of nephroblastoma is 7.6 cases per 1,000,000 children younger than 15 years of age and 1 case per 10,000 infants [5]. In 2001, Humberto reported that despite majority of children in the USA who are treated with nephroblastoma get cured, the disease still affects over 400 children per year [6]. Also, Aronson et al. in 2014 reported the incidence of nephroblastoma in South Africa of 5.4 per 1,000,000 children below 15 years [7]. In a study by Okello et al. which was done in Uganda. it was reported that the magnitude of nephroblastoma was spanning at approximately 6.8% of childhood malignancies [8].

The pathogenesis of nephroblastoma like any other malignancies is conservatively linked with mutation of various genes and other genomic components. Several genetic aberrations have been recognized with a role in pathogenesis and poor outcome of nephroblastoma including aberrations in WT1 gene mapped on 11p13, WT2 gene mapped on 11p15, and loss of heterozygosity of 16q and 1p [9–11]. In addition, inactivation of the P53 gene is also found particularly in anaplastic tumors and in tumors with focal anaplasia may only be inactivated in anaplastic areas [1, 12]. Genetic changes resulting in loss of P53 function are also the commonest change observed between primary and relapse tumor samples from patients with nephroblastoma [13]. Several studies have shown that detection of p53 by immunostaining is strongly associated with anaplasia in nephroblastoma [14, 15].

The tumor suppressor gene p53 has been recognized as one of the most important cancer-related genes. Mutations in p53 are causally involved in tumorigenesis or tumor progression, and it has been reported to be associated with aggressiveness and poor prognosis of many human cancers [9, 12, 14]. It is well known that a functional p53 protein has been linked to cell cycle checkpoint control, and the presence of p53 mutations may contribute to the increased DNA content and the irregular mitotic figures observed in anaplastic nephroblastoma [1]. Moreover, the importance of p53 in directing cells with DNA damage into an apoptotic pathway may provide a molecular basis for the insensitivity of anaplastic tumors to therapeutic intervention [16]. Several studies have been carried out to determine the role of p53 protein expression as a prognostic factor in patients with nephroblastoma. Most of these studies have established a strong association between p53 overexpression and anaplasia in nephroblastoma [9, 14, 17].

This study aims to determine the level of immunohistochemical expression of P53 protein and its associated factors in formalin-fixed paraffin-embedded (FFPE) tissue blocks of patients diagnosed with nephroblastoma.

## Materials and methods

### Study design and setting

This was a cross-sectional analytical laboratory-based study. The study was conducted in the Department of Pathology at Makerere University College of Health Science in Kampala, Uganda. The department is located within Mulago National Referral Hospital which is the only national referral hospital where there is also cancer treatment center (Uganda Cancer Institute) which provides oncology services including pediatric oncology services for Ugandans as well as patients from neighboring countries such as Democratic Republic of Congo (DRC), Rwanda, South Sudan, and Somalia.

### Patients’ characteristics and recruitment process

We included in the analysis data of patients who were diagnosed previously with nephroblastoma from January 2012 to December 2015. Retrospective collection of the required clinical data of the patients and retrieval of the formalin fixed paraffin-embedded tissue blocks of the patients were done followed by histological re-evaluation of the previous diagnosis. All cases with complete clinical information and available FFPE tissue block of good quality were included in the analysis. But all cases with incomplete clinical data, missing FFPE tissue blocks, and spoilt FFPE tissue blocks by insects were all excluded from the analysis.

### Sampling method

A total of 83 cases with confirmed histological diagnosis of nephroblastoma were included in the analysis. Sampling of the cases was done conveniently, and all cases that met the inclusion criteria were selected.

### Hematoxylin and eosin staining

For re-evaluation of the previous histological diagnosis, all of the FFPE tissue blocks of the selected cases were sectioned at the thickness of 4 μm and stained with hematoxylin and eosin stains as previous [18]. Tumor staging as well as risk stratification of the cases was done based on the International Society of Paediatric Oncology (SIOP) [19].

### Immunohistochemical staining for immunohistochemical expression of P53 protein

The FFPE tissue blocks were sectioned at 4 μm thickness and placed on poly-l-lysine-coated glass slides and placed on a hot plate for dewaxing for 10 min followed by being brought down to distilled water and then rinsed in phosphate tris buffer (PTB) solution. Endogenous peroxidase activity was blocked by placing the sections in 3% H_2_O_2_ for 5 min and rinsed 3 times in distilled water. The slides were then incubated with DO-7 antihuman p53 monoclonal primary antibody for 60 min at room temperature followed by two 5-min washes in PTB solution. The sections were incubated with biotinylated link anti-mouse immunoglobulin for 30 min and washed twice in PTB solution, followed by incubating with streptavidin–biotin-horseradish peroxidase complex (DAKO LSAB 2 system) for 30 min. The charged glass slides were washed in PTB solution for 45 min and stained in diaminobenzidine for visualization of antigen antibody binding. Finally, the sections were counterstained with hematoxylin for immunohistochemical evaluation. Considering tumor heterogeneity which is more likely to affect positivity of a given antibody, we stained at least four tissue blocks for every case.

### Assessment of immunohistochemical expression of P53 protein

The slides were evaluated using light microscopy by two experienced pathologists independently, and scoring for positivity and intensity of immunostaining of the tumor cells was done according to a previous study [20]. In cases where the two pathologists disagreed, a third opinion from the third pathologist (tie breaker) was sought, and final decision was made by obtaining two similar interpretations. For assessment of p53 immunostaining, tumor cells with clearly brown reaction in the nuclei were counted by monitoring at least 1000 tumor cells from more than 5 high-power fields (HPFs) where positive cells were present at relatively uniform density, and the percentage was then calculated. The density score for the percentage of tumor cells with nuclear positivity was quantified as follows: 0:1–25%, 1:26–50%, 2:51–75%, and 3: > 75%. The numbers of immunopositive cells were counted, and the case was categorized as negative when none or only a few (< 5%) cells on the whole slide showed weak staining [17]. Then intensity of nuclear staining of the tumor cells was graded as follows: weak (light brown color staining), 1; moderate staining (moderate brown color staining), 2; and strong (dark brown color staining), 3. Density and intensity scores were combined and converted to a third score. All stains with an intensity and density score higher than 2 received a score of 1 (positive cases), and other cases with a combination of density and intensity score less than 2 were considered to be negative [20].

### Data collection and research tool

Information regarding clinical data on age of the patients, sex, lag period, tumor size, tumor laterality, preoperative chemotherapy, and clinical presentation was extracted from the patients’ files as well as patients’ laboratory requisition forms. Also, histological components (anaplasia, blastemal, and epithelial), status for lymph node and surgical margins, and blood vessel and ureter involvement by tumor vessels were evaluated from the prepared FFPE tissue sections and recorded in the self-designed data collection form.

### Data analysis

Data collected were edited and cleaned for missing data and other errors by running frequency tables and crosstabs. Analysis was performed using STATA version 12.0. All categorical variables were presented in frequencies and percentages, and continuous variables were summarized in mean ± standard deviation (SD) and also median (interquartile range (IQR)). Binary logistic regression analysis was performed to obtain the independent predictors of immunohistochemical expression of P53 protein. Only variables with *p* < 0.05 in univariate regression analysis were carried to multivariable regression analysis to determine factors that would independently predict the expression of P53 after adjusting for possible confounders. Adjusted odds ratios (AORs) were calculated at 95% confidence interval (CI) in multivariable regression analysis. A two-tailed *p* < 0.05 was considered statistically significant.

## Results

Table 1 presents the demographic and clinical characteristics of the patients. A total of 83 nephrectomies of patients with nephroblastoma were included in the present study. The mean age of the patients was 4.3 ± 0.9 years (range: 3–7 years). Females comprised majority (62.7%, *n* = 52) of all the patients. Majority (67.5%, *n* = 56) of the patients were taken to hospital for medical services after a period of more than 3 months since onset of signs and symptoms. Also, most (42.2%, *n* = 35) of the patients had advanced tumor stage (III–V), and majority (74.7%, *n* = 62) had undergone chemotherapy prior nephrectomy, and (10.8%, *n* = 9) children died before being discharged.

**Table 1:** Demographics and clinical characteristics of the patients (*N* = 83)

Variables	Frequency (*n*)	Percentage (%)
**Age (years)**		
≤ 4	49	59.0
> 4	34	41.0
**Female**		
Male	31	37.3
Female	52	62.7
**Tumor laterality**		
Right	45	54.2
Left	38	45.8
Bilateral	-	-
**SIOP clinical stage**		
I	35	42.2
II	13	15.7
III	21	25.3
IV	10	12.0
V	4	4.8
**Tumor size (cm)**		
≤ 4	20	24.1
> 4	63	75.9
**Preoperative chemotherapy**		
Yes	62	74.7
No	21	25.3
**Lag period (months)**		
≤ 3	27	32.5
> 3	56	67.5
**Clinical outcome**		
Alive	68	81.9
Dead	15	18.1
**Side effects of chemotherapy**		
Nausea and vomiting	47	56.6
Loss of hair	6	7.2
Anemia	30	36.2

### Histopathologic evaluation and immunohistochemical expression of P53 protein

**Fig. 1:**
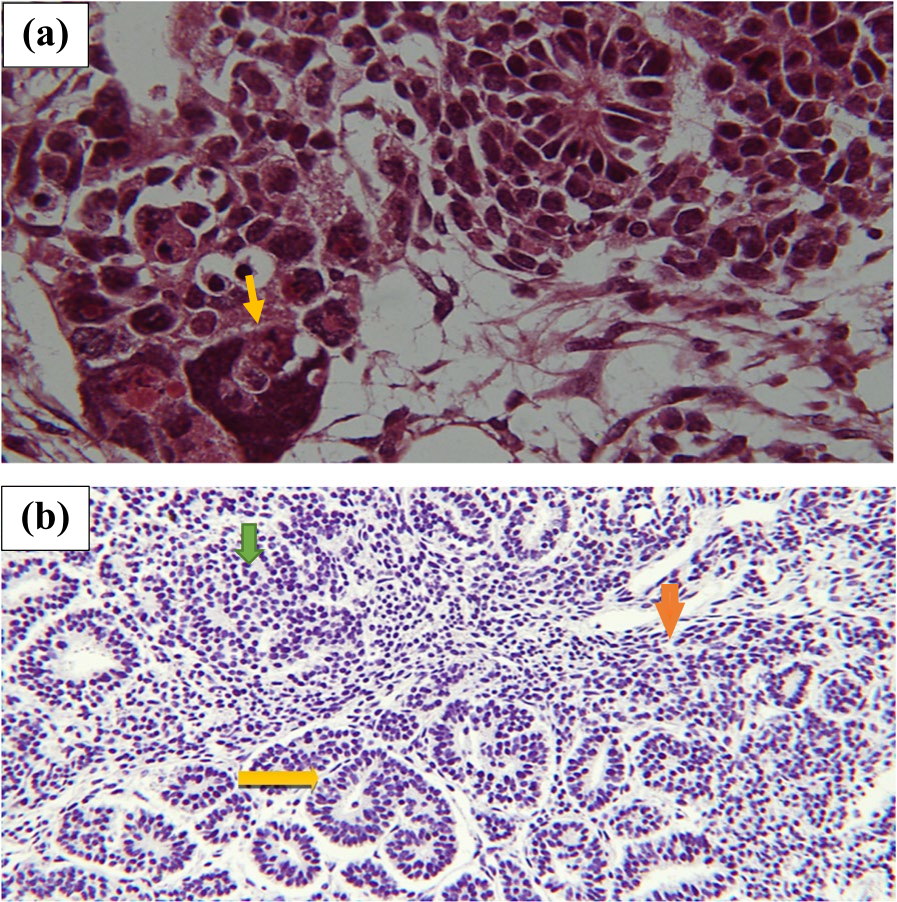
**a** Photomicrograph showing anaplastic tumor cells with multipolar mitosis (arrow) (hematoxylin and eosin stain, × 200) and **b** photomicrograph showing a classical triphasic case of nephroblastoma with blastemal pattern (green arrow), epithelial pattern (yellow arrow), and stromal pattern (orange arrow) components well represented (hematoxylin and eosin stain, × 100)

**Fig. 2:**
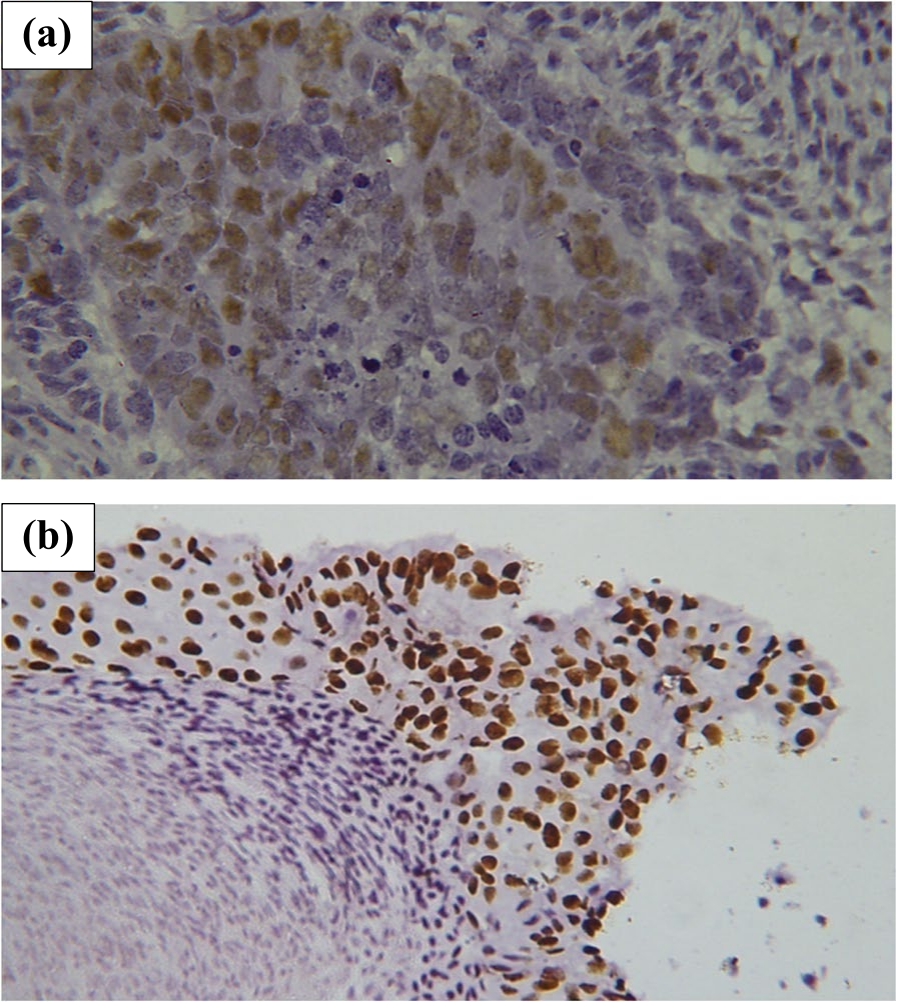
**a** Photomicrograph showing strong intranuclear immunoreactivity for P53 protein for a case of nephroblastoma with anaplasia (immunohistochemistry stain for P53 antibody, × 200) and **b** photomicrograph of showing strong intranuclear immunoreactivity for P53 protein for a case with predominant blastemal component (immunohistochemistry stain for P53 antibody, × 200)

The vast majority (81.9%, *n* = 68) of the cases were triphasic (Fig. 1a) followed by blastemal morphologic pattern which was observed in (11%, *n* = 9) of all the cases. Also, almost one-quarter (21.7%, *n* = 18) of the cases were in high-risk group. Concerning histologic favorability, it was observed that majority (93%, *n* = 77) of the cases were of favorable histological type (non-anaplastic), and the remaining (7%, *n* = 6) cases were of unfavorable histological type (anaplastic) (Fig. 1b). Immunohistochemical expression of P53 (Fig. 2a and b) protein was found in (8.4%, *n* = 7) of all the cases, and high rate of the expression of the P53 protein was observed in anaplastic cases (83%, *n* = 5) and those with blastemal morphologic pattern (33%, *n* = 3) (Table 2).

**Table 2:** Histopathologic features and immunohistochemical expression of P53 (*N* = 83)

Histopathologic features	Frequency (*n*)	Percentage (%)
**Risk stratification**		
Low risk	36	43.4
Intermediate risk	29	34.9
High risk	18	21.7
**Morphologic patterns**		
Blastemal	9	10.9
Epithelial	2	2.4
Stromal	1	1.2
Blastemal + stromal	2	2.4
Blastemal + epithelial	1	1.2
Triphasic	68	81.9
**Presence of anaplasia**		
Yes	6	7.2
No	77	92.8
**P53 expression**		
Positive	7	8.4
Negative	76	91.6

### Predictors of immunohistochemical expression of P53 protein

Table 3 presents the binary logistic regression analysis for the predictors of immunohistochemical expression of P53 protein. Under univariate logistic regression analysis, being male (*UOR* = 1.13, 95% *CI* = 0.65–3.43, *p* = 0.872), having advanced tumor stage (stages III–V) (*UOR* = 1.34, 95% *CI* = 0.29–4.95, *p* = 0.072), and having tumor size of greater than 4 cm (*UOR* = 1.27, 95% *CI* = 0.68–5.08, *p* = 0.493) all showed increased risk for the expression of P53 protein compared with their counterparts, but the difference did not reach statistical difference.

**Table 3:** Multivariable logistic regression analysis for predictors of immunohistochemical expression of P53

	**P53 expression**		**Univariate logistic regression analysis**	**Multivariable logistic regression analysis**
**Variables**	**Positive**	**Negative**	**UOR (95% *CI*), *P***	**AOR (95% *CI*), *P***
**Age (years)**				
≤ 4	1	48	1.00	1.00
> 4	6	28	3.47 (0.74–0.91), 0.041	1.39 (0.58–6.12), 0.703
**Sex**				
Male	3	28	1.13 (0.65–3.43), 0.872	
Female	4	48	1.00	-
**Lag period (months)**				
≤ 3	4	23	1.00	-
> 3	3	53	0.46 (0.85–13.07), 0.704	
**SIOP clinical stage**				
I–II	3	45	1.00	-
III–V	4	31	1.34 (0.29–4.95), 0.072	
**Tumor size (cm)**				
≤ 4	3	17	1.00	-
> 4	4	59	1.27 (0.68–5.08), 0.493	
**Risk stratification**				
Low	1	35	1.00	1.00
Intermediate	1	28	0.31 (0.78–13.07), 0.511	-
High	5	13	1.94 (3.60–11.24), 0.001	3.42 (7.91–12.55), 0.037
**Morphologic pattern**				
Other patterns	0	6	1.00	1.00
Blastemal	3	6	1.39 (2.55–21.90), 0.044	1.1 (0.674–5.12), 0.201
Triphasic	3	65	1.72 (7.41–15.06), 0.023	1.33 (0.56–5.89), 0.812
**Presence of anaplasia**				
Yes	5	1	2.03 (4.05–9.69), 0.025	1.41 (13.85–4.46), 0.001
No	2	75	1.00	1.00

Independent factors were adjusted for age, risk, morphologic pattern, and anaplasia in multiple logistic regression analysis

When independent variables that were fitted into multivariable logistic regression were adjusted for all factors, only high risk and anaplasia remained the predictors of immunohistochemical expression of P53 protein. High-risk cases were 3.42 times more likely to express P53 compared with fold increased risk of expressing P53 protein for cases in high-risk group compared with low risk, and the difference between the two compared groups reached statistical significance (*AOR* = 3.42, 95% *CI* = 7.91–12.55, *p* = 0.037). Also, cases with anaplastic changes were 1.42 times more likely to express P53 than non-anaplastic cases, and that difference was significant (*AOR* = 1.41, 95% *CI* = 13.85–4.46, *p* = 0.001).

Other factors include being aged more than 4 years (*AOR* = 1.39, 95% *CI* = 0.583–6.116, *p* = 0.703) and having triphasic morphologic pattern (*AOR* = 1.33, 95% *CI* = 0.556–5.894, *p* = 0.812) even though they showed increased risk for P53 expression compared to their counterparts; however, the difference did not reach statistical difference.

## Discussion

This study was conducted with the aim of determining the immunohistochemical expression of p53 protein and its predictors from nephrectomy specimens of patients with nephroblastoma. The main findings of this study included the following: unfavorable histology (anaplastic changes) as well as high-risk group was significantly associated with immunohistochemical expression of the P53 protein.

There is a strong association between immunohistochemical expression of the P53 protein and mutation of P53 gene [21], which explains as one of the main pathogenic mechanism for the development of nephroblastoma despite the discrepancies in the immunohistochemical detection rates of the P53 protein in various studies that have been reported in the literature. However, it should be understood that mutation of the P53 gene is not the sole pathogenic mechanism for initiation and even progression of nephroblastoma, which may help to explain the reason in situations when there is either very low rate of immunohistochemical detection of the protein or sometimes not all [15, 17]. This may also be justified by the concept of tumor heterogeneity which usually necessitates sampling of as many as possible number of tissue blocks so as to increase the probability of detecting the P53 protein when using immunohistochemistry method.

In respect to the immunohistochemical expression of the P53 protein in our study, we observed that 8.4% out of all the 83 cases was positive. This level of expression is close to 9.7% and 8.6% of the immunohistochemical detection rate of P53 protein in nephroblastoma which was reported in the India [22] and South Africa [17], respectively. However, the level of immunohistochemical expression of P53 protein in the present study was lower than 16.7% and 16.0% of the immunohistochemical expression of P53 which was reported in Nigeria [14] and India [23], respectively. Other studies which were done in Egypt reported 56% [15] and 60.3% [24] percentages of immunohistochemical expression of P53 among patients with nephroblastoma, which are quite higher than the percentage of P53 immunohistochemistry expression observed in the present study.

There are number of factors which can explain the discrepancy in prevalence of immunohistochemical detection rate of P53 protein in patients with nephroblastoma from different studies. Variation in the methods of scoring positivity for the P53 protein by means of immunohistochemistry may explain the difference in the immunohistochemical detection rates. For example, the two previous studies which were both conducted in Egypt by Salama et al. [24] and Darwish et al. [15] which both reported significantly high prevalence of P53 protein expression used a cut-off point of 5%, implying that all cases which showed expression of 5% and more were considered to be positive. This directly influenced the rate of positivity by inflating it. Also, the ability to identify cases with anaplasia in the different studies may also account for the difference in the immunohistochemical detection of P53 protein, because the number of anaplastic cases in a given study determines the detection rate of P3 expression as it has been reported that anaplastic cases have high rate of expressing the P53 protein compared to non-anaplastic cases [14, 15, 17, 22]. This is why it is highly recommended to increase the number of sections per case for the purpose of increasing the ability to detect cases with anaplastic changes [17]. Additionally, it must also be understood that the percentage of anaplastic nephroblastoma ranges from 5 to 10% [25], indicating that it is not so high.

In this study, we found a strong association between immunohistochemical expression of P53 protein and anaplasia which is in agreement with the findings in other studies which also found that the presence of anaplasia in nephroblastoma was significantly associated with high level of immunohistochemical expression of the P53 protein [14, 15, 22, 24]. In another study by Percicote et al. reported quite a high mean of immunohistochemical expression of P53 protein in cases with unfavorable histology compared with cases with favorable histology [26]. In this study, p53 expression in tumors with unfavorable histology was present mainly in anaplastic foci of the tumor with scattered non-anaplastic cell positivity. However, in one study, it was reported that the immunohistochemical expression of the P53 protein was marked even in non-anaplastic foci of the tumor sections examined in the study [17] which is different from our observation.

These discrepancies may be attributed partly due to methodological differences and also experience of the reporting pathologists in identifying anaplastic cells microscopically. Also, there is a possibility that genetic variation among patients from different races could also explain the genetical influence in context of P53 gene mutations as reflected by immunohistochemical expression of the protein [27]. Handling of the nephrectomy specimens in the pre-analytical phase which involves both optimal fixation and timely fixation has great contribution in determining the immunohistochemical expression of the P53 protein [28].

There was also a positive association of high-risk cases with expression of P53 protein in our study which is similar to the findings in the studies of Salama et al. [24] and Darwish et al. [15]. Both studies found that P53 protein was immunohistochemically highly expressed compared with cases in low-risk group. This is because high-risk group of nephroblastoma consists mainly of blastemal and anaplastic morphological patterns of nephroblastoma which both bear a high rate of expression of P53 [22]. Therefore, it is very important to ensure that the right morphological pattern is assigned for nephroblastoma cases during histological evaluation of the submitted specimens so as to avoid such histological features which carry high risk with poorest prognosis especially when not managed appropriately.

### Strengths and limitations of the study

This study reports on immunohistochemical expression of P53 protein using relatively a large sample size compared to many previous studies; this increased the power of the study and strength to the conclusions that were drawn regarding the results. However, there were a number of methodological limitations which our study encountered among which include inability to include analysis of P53 gene mutations due to insufficient funds and lack of follow-up data which could have enabled us to analyze survival of the patients. Another limitation is that the data used in this analysis were from a single hospital setting which makes not possible to be able to generalize the results.

## Conclusion

The immunohistochemical expression of P53 protein in our study was significantly marked in cases with unfavorable histology (anaplastic) followed by cases with blastemal morphologic pattern. There was significant association of high-risk group and being anaplastic with immunohistochemical expression of the P53 protein. Future prospective studies may focus on the other aberrations of the P53 gene rather than its mutations as it has been the case for most of previous studies including the present study which investigated immunohistochemical expression of the P53 protein which usually is an indicator of mutation of the P53 gene.

## Data Availability

The dataset used for this study is restricted by the institution review board of the institution due to containing sensitive patient information. However, it can only be accessed upon reasonable request from the Directorate of Research and Graduate Training (DRGT), Makerere University, P. O. Box 7062, Kampala, Uganda, drgt@mak.ac.ug.
